# Identifying the Cause of Toxicity of a Saline Mine Water

**DOI:** 10.1371/journal.pone.0106857

**Published:** 2014-09-02

**Authors:** Rick A. van Dam, Andrew J. Harford, Simon A. Lunn, Marthe M. Gagnon

**Affiliations:** 1 Environmental Research Institute of the Supervising Scientist, Department of the Environment, Darwin, Northern Territory, Australia; 2 Department of Environment and Agriculture, Curtin University, Perth, Western Australia, Australia; Northwest Fisheries Science Center, NOAA Fisheries, United States of America

## Abstract

Elevated major ions (or salinity) are recognised as being a key contributor to the toxicity of many mine waste waters but the complex interactions between the major ions and large inter-species variability in response to salinity, make it difficult to relate toxicity to causal factors. This study aimed to determine if the toxicity of a typical saline seepage water was solely due to its major ion constituents; and determine which major ions were the leading contributors to the toxicity. Standardised toxicity tests using two tropical freshwater species *Chlorella* sp. (alga) and *Moinodaphnia macleayi* (cladoceran) were used to compare the toxicity of 1) mine and synthetic seepage water; 2) key major ions (e.g. Na, Cl, SO_4_ and HCO_3_); 3) synthetic seepage water that were modified by excluding key major ions. For *Chlorella* sp., the toxicity of the seepage water was not solely due to its major ion concentrations because there were differences in effects caused by the mine seepage and synthetic seepage. However, for *M. macleayi* this hypothesis was supported because similar effects caused by mine seepage and synthetic seepage. Sulfate was identified as a major ion that could predict the toxicity of the synthetic waters, which might be expected as it was the dominant major ion in the seepage water. However, sulfate was not the primary cause of toxicity in the seepage water and electrical conductivity was a better predictor of effects. Ultimately, the results show that specific major ions do not clearly drive the toxicity of saline seepage waters and the effects are probably due to the electrical conductivity of the mine waste waters.

## Introduction

In addition to low pH and elevated concentrations of trace metals and metalloids such as Al, As, Cu, Cd, Ni and Zn, elevated salinity (also referred to as Total Dissolved Solids [TDS] or Electrical Conductivity [EC]), is recognised as being a key contributor to the toxicity of many mine waste waters [1], [2]. Salinity represents an integrated measure of the concentrations of major ions, namely Na, Ca, Mg, K, Cl, SO_4_ and HCO_3_, all of which are biologically essential and generally viewed to possess low toxicity. Historically, salinity was viewed as a natural stressor as opposed to a toxicant and, accordingly, the role of salinity in effluent toxicity was rarely considered [3]. However, the past two decades have seen an increasing focus on the importance of salinity in causing or contributing to effluent toxicity. Whilst there is an improved understanding of the toxicity of major ions and their relative importance in determining salinity toxicity, [4], [5], the complex interactions between the major ions (in terms of speciation and competition), and large inter-species variability in response to salinity, make it difficult to relate toxicity to causal factors in specific saline waters [6].

Salinity can directly affect freshwater organisms by two primary means: (i) osmotic stress related to the combined major ion concentrations (i.e. a general salinity effect); and/or (ii) toxicity of specific major ions or ion combinations. McCulloch et al. [3] outlined methods to separate toxicities due to salinity or specific ions using Toxicity Identification Evaluation (TIE) approaches with three main objectives: 1) characterise the chemical properties of the toxicant; 2) identify the specific toxicant or compounds causing toxicity and; 3) determine whether the identified compound is causing the observed toxicity in the effluent. Such approaches have been utilised with some success for mining and other industrial effluents [4], [6], [7].

The Savannah Nickel Mines (SNM), located in north-west Western Australia, approximately 700 km south-west of Darwin, is an operating underground (formerly open cut) mining and milling operation with associated tailings and water storage facilities. The ore body is associated with a palaeo-proterozoic mafic/ultramafic magma conduit and the nickel-copper-cobalt rich sulfide mineralisation has developed around the MgO rich ores. The region experiences a tropical semi-arid climate with distinct dry (April–November) and wet (December–March) seasons, and an average annual rainfall of 555 mm. Magnesium sulfate (MgSO_4_) rich seepage waters high in TDS flow from the toe of a water storage facility, into Mine Creek, an ephemeral 1^st^ order creek located on the SNM mining lease. This creek discharges into Fletcher Creek in the middle of the catchment where it is a 2^nd^ order creek and has a low <2% gradient. Fletcher Creek is a significant tributary of the upper Ord River with a catchment area of 530 km^2^ and a discharge of up to 198 GL/y during high-rainfall years influenced by cyclones. The land in the catchment is uncleared rangelands dominated by spinifex and low, open eucalypt wood and is primarily used for grazing cattle. During the dry season, the seepage is pumped back to site and there is negligible surface flow to Fletcher Creek. However, for periods during the wet season (typically hours to days), high rainfall and discharge result in a mix of seepage and surface flow in Mine Creek discharging into Fletcher Creek. This is of most potential concern during the late wet/early dry season, when biological diversity is high and the recessional surface flow conditions reduce the extent of dilution of the seepage water.

A toxicity assessment of the seepage water (EC–2300 µS/cm) found that it caused significant adverse effects to two of five freshwater species, namely the green alga, *Chlorella* sp., and the cladoceran, *Moinodaphnia macleayi* [8]. Whilst previous studies did not definitively identify the chemical constituents causing the observed toxicity, the absence of elevated concentrations of toxic trace metals led to the hypothesis that the observed effects were due to the elevated major ion concentrations (mostly MgSO_4_) resulting in an ion imbalance that leads to osmotic stress (i.e. a salinity effect). However, Fletcher Creek upstream of the mine often had low SO_4_ but high concentrations of HCO_3_ and EC could not be used as a surrogate measure to monitor the impacts of the seepage water on Fletcher Creek. The present study aimed to address the hypothesis that the effects were due to osmotic stress, with the following key aims:

- confirm the extent of toxicity of seepage water to *Chlorella* sp. and *M. macleayi*;
- assess whether the toxicity of the seepage water is solely due to its major ion constituents; and
- if toxicity was related to ion concentrations, determine which major ions are the leading contributor to the seepage toxicity.

Methods similar to those described by McCullough et al. [3] and Goodfellow et al. [6] were used to attempt to identify the source of toxicity. In particular, the toxicity of mine seepage (MS) was compared to the toxicity of a synthetic seepage (SS) comprising only the major ion composition of MS.

## Materials and Methods

### 2.1 General laboratory procedures

The Northern Land Council granted permission for water collections at Bowerbird Billabong (latitude 12° 46′ 15″, longitude 133° 02′ 20″). Panoramic Resources approved the collection of water samples from Savannah Nickel Mine (latitude 17° 21′ 21″, longitude 128° 01′ 50″). No endangered or protected organisms were used in this study and ethics approval was not required for *M. macleayi* because it is zooplankton.

All equipment used for solution preparation and toxicity testing was made of chemically inert materials (e.g. Teflon, glass or polyethylene). All plastic and glassware was soaked in 5% nitric acid for 24 h before undergoing a detergent wash (Gallay Clean A powder, Gallay Scientific, Burwood, Australia) and rinsed in a laboratory dishwasher using reverse osmosis (RO) water. All glassware (except volumetric flasks) was silanised with 2% dimethyldichlorosilane in 1,1,1-trichloroethane (Coatasil, AJAX, Seven Hills, Australia,) to reduce metal adsorption to the glass. All reagents used were analytical grade and stock solutions were made up in Milli-Q water (18 Ω/cm, Millipore Ltd, Billerica, MA, USA).

### 2.2 Water collection and preparation

#### 2.2.1 Natural waters

Mine seepage (MS) samples (20 L) from the toe of the water storage facility were collected from SNM on two separate occasions, on 15 June and 16 September 2011. After an initial test to assess the suitability of various control waters (data not shown), Magela Creek Water (MCW) was selected as the control/diluent water for the duration of the project. The MCW was collected monthly from Bowerbird Billabong (latitude 12° 46′ 15″, longitude 133° 02′ 20″), a site on Magela Creek, Northern Territory. Magela Creek water typically is slightly acidic (pH ∼5.5–6.5), and has very low electrical conductivity (∼5–15 µS/cm), water hardness (∼3–6 mg/L as CaCO_3_), alkalinity (5–10 mg/L as CaCO_3_), and concentrations of trace metals. All MS and MCW samples were transported to the laboratory on the same day of collection. Upon arrival at the laboratory, samples were filtered with a peristaltic pump (Gamet, Armidale, Australia) using 3 µm filters (Sartorius Sartopure PP2 depth 3 µm Midicap, Goettingen, Germany), then refrigerated at 4°C until test commencement.

#### 2.2.2 Synthetic seepage

Synthetic Seepage was prepared to simulate the major ion composition of MS (Table 1). All chemicals used to make the SS were of Analytical Grade. The SS solutions were prepared by weighing chemicals on an analytic balance (R200D, Sartorius, Geoettingen, Germany) and dissolving the predetermined amounts into the MCW diluent. If necessary, stock solutions of the chemicals were prepared first, with the required volume then pipetted into the MCW diluent. The minerally dilute MCW was used in favour of deionised water because one of the test species, *M. macleayi*, cannot be cultured or tested in synthetic waters prepared from deionised water [9]. Dissolution of the separate salts was encouraged through stirring and, if necessary, mild warming (i.e. <50°C). The water was left for no longer than 3 d and was not removed from the stirrer until all the salts had been visibly dissolved. In most cases dissolution occurred within a few hours.

**Table 1:** Key physico-chemistry and major ion composition of control/diluent and mine and synthetic seepage waters.

Analyte	Water type				
	Control/diluent	Seepage sample 1(15 June 2011)		Seepage sample 2(16 Sept. 2011)	
		Mine	Synthetic	Mine	Synthetic
pH	5.8–6.4	7.6	7.7	8.0	8.0
ElectricalConductivity (µS/cm)	21–54	2320	2390	3090	3060
Dissolved organiccarbon (mg/L)	1.0–1.6	0.8	1.0	1.3	1.6
Calcium(Ca, mg/L)	<0.5–0.2	240	210	320	320
Potassium(K, mg/L)	<0.5–0.3	8.3	9.4	10	11
Magnesium(Mg, mg/L)	0.9	200	190	240	250
Sodium (Na, mg/L)	1.1	84	86	96	97
Chloride (Cl, mg/L)	1.6–2.0	21	25	24	22
Carbonate(HCO_3_, mg/L)	6.0–11	210	170	210	170
Sulfate (SO_4_, mg/L)	<0.5	1300	1200	1800	1900

#### 2.2.3 Toxicity test solutions

Test solutions were prepared by diluting MS, SS or individual salt solutions with a clean diluent (MCW). The pH was then adjusted, if necessary, using 0.02 M HNO_3_ or 0.0125 M NaOH. Test solutions were prepared in bulk at the start of a test in 1 or 5 L polyethylene screw-capped containers and refrigerated (4°C) until required.

### 2.3 Toxicity testing – *Chlorella* sp

#### 2.3.1 Culture

An algal culture was maintained in MBL medium [10] at 29±1°C on a 12∶12 h photoperiod (Philips TL 40 W cool white fluorescent lighting; 100–150 mol/m^2^/s). Tests were conducted using exponentially growing cells extracted from a culture which was 4 to 5-d-old [11].

#### 2.3.2 General test method

*Chlorella* sp. growth inhibition, measured as number of cell doublings/day, was assessed following exposure to the test waters for 72 h [9]. At the commencement of each test, algal cells were selected from a 4 to 5-d old culture, rinsed in Milli-Q water using centrifugation, resuspended in 10–15 mL of diluent, which was then passed through an electronic particle counter (see below) to determine the cell density. The volume required to give each flask a starting cell density of 3×10^4^ cells/mL was dispensed into each replicate, which consisted of a 100 mL silanised borosilicate glass Erlenmeyer flask containing 30 mL of pre-warmed test solution, covered with an aluminium foil lid. The flasks were randomly placed in an environmental cabinet and incubated at 29±1°C on a 12∶12 h photoperiod at 100–150 µmol photons PAR/m^2^/s. After 48 h and 72 h, a 2 mL sample of each solution was extracted for cell enumeration (see below). After 72 h the pH, EC and dissolved oxygen (DO) of the solutions were measured and the test was terminated.

Cell counts were determined using either an electronic particle counter (Coulter Counter, MS3, Beckman Coulter, Jersey USA) or enumerated manually using a haemocytometer and microscope. From these cell counts, cell densities were calculated for each treatment at 48 and 72 h and, finally, the growth rate was determined from the cell densities using linear regression. A regression line was plotted for log10 cell density v time (h) to determine the slope of the line for each flask, which is equivalent to the cell division rate per hour (µ) for each treatment. Daily doubling times were calculated by multiplying this value µ×24×3.32 (constant) and were statistically compared (see *Statistical Analysis*).

Tests were considered valid if they met the following acceptability criteria: the growth rate of the control algae was within the range 1.4±0.3 doublings/day; the percent co-efficient of variation (%CV) of the control growth rate was <20%; and the pH, EC and DO of the control treatments varied by <20% over the test duration [9]. Additionally, measured nitrate (NO_3_^−^) and phosphate (PO_4_^3–^) concentrations were checked against nominal concentrations, while measured ammonia (NH_4_^+^) was checked to ensure it did not exceed 0.2 mg/L in any treatment.

#### 2.3.3 Test details

Comparisons of the toxicity of MS and SS to *Chlorella* sp. were undertaken for both of the MS samples collected. Test dilutions of MS and SS were 0, 12.5, 25, 50 and 100% (in MCW) for all tests. The toxicity to *Chlorella* sp of three single salts, namely NaCl (3.0, 99, 260, 390, 550, 780, 1120, 1610, 220, 3200, 3600 and 4200 mg/L; measured; 2 tests, data pooled), NaHCO_3_ (as HCO_3_–4.0, 170, 300, 620, 1200 and 2500 mg/L; measured), was assessed in order to better understand the alga’s responses to the seepage waters. Finally, an assessment of the toxicity of undiluted SS to which three metals, bromine (Br; 220 µg/L), manganese (Mn; 440 µg/L) and strontium (Sr; 1400 µg/L) had been added, to *Chlorella* sp., was compared to that of control water, unmodified SS and MS (second sample) to determine if these metals may have been contributing to MS toxicity. For this test, microscopy was used in addition to electronic particle counting to enumerate and observe algal cells, in order to assess the influence of solute precipitation and particle aggregation in high salinity solutions on electronic particle counting results.

### 2.4. Toxicity testing – *Moinodaphnia macleayi*

#### 2.4.1 Culture

Individuals of *M. macleayi* were kept in small vials (45 mL plastic vials with snap-on lids, lids have two air-holes) with 30 mL aliquots of MCW, replaced daily. Individuals were fed FFV (Fermented Food with Vitamins; 1 µL/mL) and algae (*Chlorella* sp. at 2×10^5^ cells/mL) daily and were kept in incubators at 27±1°C on a 12∶12 h photoperiod. A total of 24–36 individuals were maintained at all times, with further individuals maintained depending on experimental demands. Every 4 to 5 d, second brood neonates were collected to restart the stock.

#### 2.4.2 General test method

*Moinodaphnia macleayi* reproductive impairment, measured as the total number of offspring per adult, was assessed following exposure to the test waters for 3 reproductive broods (∼6 d) [9]. At the commencement of each test, suitable *M. macleayi* neonates (i.e. <6-h old) were collected from adult cultures and randomly pooled together in a crystallising dish. Neonates were selected from the dishes and placed individually into 45 mL plastic test vials containing 30 mL of sample water and food (30 µL of FFV and 2×10^5^ cells/mL of *Chlorella* sp.). There were 10 replicate vials for each treatment. The vials were then randomly placed on a tray and the trays randomly placed in an environmental cabinet and incubated at a temperature of 27±1°C with a 12∶12 h photoperiod.

Each day, observations on the health of the female, the number of neonates produced and the number of surviving neonates, were recorded. Following these observations, each individual adult cladoceran was transferred to a new vial with new test water and food. The pH, EC and DO of both the old and new solutions were measured daily. The test was terminated when ≥80% of the control cladocerans had successfully produced their third brood (∼6 d). The mean number of offspring per adult was summarised for each treatment and statistically compared (see *Statistical analysis*).

Tests were considered valid if they met the following acceptability criteria: reproduction in the control averaged 30 or more surviving neonates per female; survival in the control was ≥80%; and the pH, EC and DO of the control treatments varied by <20% over the test duration [9].

#### 2.4.3 Test details

Comparison of the toxicity of MS and SS to *M. macleayi* was undertaken only for the second MS sample collected. Test dilutions of MS and SS were 0, 12.5, 25, 50 and 100% (in MCW). The toxicity of NaCl (anhydrous salt; 13, 60, 90, 100, 150, 210, 280, 410, 560, 820, 1070, 1570, 2090 µS/cm, measured) and NaSO_4_ (NaSO_4_.10H_2_0, 13, 100 200, 400, 760, 1530, 3020; µS/cm, measured) to *M. macleayi* was assessed in order to better understand the cladoceran’s responses to the seepage waters. Finally, an assessment of the toxicity to *M. macleayi* of various SS solutions from which specific major ions had been excluded was undertaken to determine if any of the major ions could be identified as contributing to seepage toxicity. Specifically, seven different SS solutions were prepared: unmodified SS; Mg excluded SS (–Mg); Ca excluded SS (–Ca); SO_4_ excluded SS (–SO_4_); and the last three SS treatments with EC adjusted using NaCl to that of the unmodified the highest EC treatment (i.e. –SO_4_, 1200 µS/cm). Where Mg and Ca were excluded, the SO_4_ counter ion was added using Na_2_SO_4_, and where the SO_4_ was excluded, the Mg and Ca counter ions were added as MgCl_2_ and CaCl_2_. The concentrations of salts required for the production of the synthetic waters were calculated using molar units. Removal of HCO_3_ from the SS resulted in a lower pH of 6.9, compared to the other solutions’ pH of 8.0. Consequently, the pH of this treatment was readjusted with NaOH to pH 8.0 on daily basis. The toxicity test with the SS solutions was conducted twice and the results pooled.

### 2.5. Chemical analyses

Chemical analysis requirements were determined on a per test basis, but were based around a common suite. The typical suite of analytes included Al, Ca, Cd, Co, Cr, Cu, Fe, K, Mg, Mn, Na, Ni, Pb, SO_4_ (inferred from S), Se, U and Zn, analysed by Inductively Coupled Plasma Optical Emission Spectroscopy (ICP-OES, Thermo Iris Intrepid 2 Radial, Waltham, USA) or Inductively Coupled Plasma Mass Spectroscopy (ICP-MS, Agilent 7500 ce, Japan), as appropriate. Carbonate (CO_3_) and/or bicarbonate (HCO_3_) were determined titrimetrically. For the second MS sample, a full ICP-MS scan of 66 elements was conducted to search for any additional elements of potential ecotoxicological concern. Trace metals and major ions were typically analysed using 0.45 µm filtered and unfiltered sub-samples, respectively. For *Chlorella* sp. tests, NO_3_, PO_4_ and NH_3_ were analysed by flow injection analysis (Lachat 8000 series).

### 2.6 Statistical analyses

Concentration-response toxicity data for both *Chlorella* sp. and *M. macleayi* (i.e. MS and SS tests, and single salt toxicity tests) were analysed using linear interpolation (CETIS, TidePool Software) in order to calculate the concentrations, and associated 95% confidence intervals, at which there were 10, 25 and 50% reductions in response compared to the controls (i.e. IC_10_, IC_25_ and IC_50_). The *M. macleayi* experiment assessing the effect of specific ion exclusion in SS was assessed using one way analysis of variance (ANOVA) on Ranks with a Dunn’s *post-hoc* test performed to determine the location of significant differences between treatment groups (α = 0.05). This ranks test was performed as the data did not meet the parametric ANOVA assumptions of normality, equal sample sizes and homoscedasticity.

In addition to the above analyses, several complementary analyses were undertaken (using Sigma Plot 11.1.0.102, System Software). Analysis of covariance (ANCOVA) was undertaken to test for differences in the concentration-response relationships between MS and SS for both species (α = 0.05). This approach compares the slopes (and y-intercept) of the linear concentration-response relationships [12]. The data did not need to be transformed for this purpose. Forward stepwise regression was performed on the reproductive response and major ion concentration data from the *M. macleayi* ion exclusion test to identify major ions that were significantly influencing the observed responses.

## Results

### 3.1 Quality control and assurance

#### 3.1.1 Chemistry

The key physico-chemistry and major ion composition of the various mine and synthetic test waters are summarised in Table 1. The major ion composition of the SS closely matched that of the MS. Notably, the second MS sample had a higher salinity than the first, due largely to seasonal factors such as tailings evapoconcentration and reduced groundwater infiltration from rainfall over the course of the dry season. The full ICP-MS scan of both MS samples indicated that Br, Mn and Sr were at relatively high concentrations of 220, 440 and 1400 µg/L, respectively.

### 3.2 Toxicity

#### 3.2.1 *Chlorella* sp

**Figure 1:**
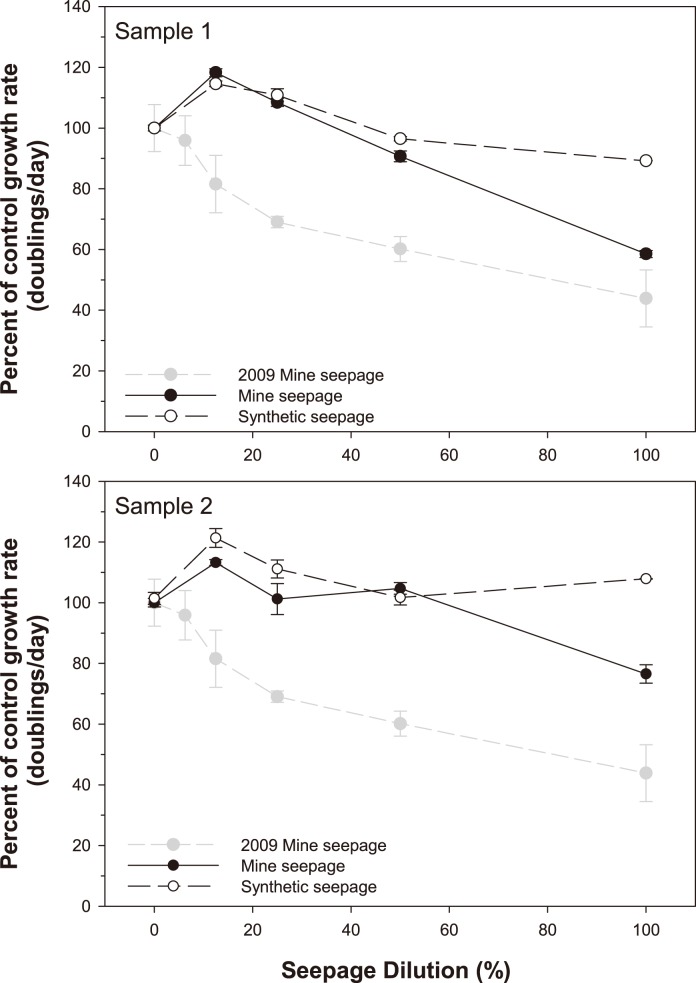
Effect of mine (MS) and synthetic (SS) seepage on growth rate of *Chlorella* sp. Results are normalized to the percent of control response, and are expressed as mean ± standard error (n = 3 per tested concentration). Mine seepage toxicity data from Harford et al. [8] are also plotted. Mean ± standard error (n = 3) control growth rates (doublings/day) for the present study were as follows: MS sample 1–1.71±0.015; SS sample 1–1.71±0.015; MS sample 2–1.59±0.023; SS sample 2–1.61±0.032.

Synthetic seepage was significantly less toxic to *Chlorella* sp. than MS for both MS samples (ANCOVA; *P* = 0.011 and *P*<0.001 for MS samples 1 and 2, respectively; Figure 1; Table 2). The differences in toxicity between SS and MS were only evident at the highest concentration (i.e. 100% seepage), with percent effects on algal growth (relative to control) exhibited at this concentration ranging from approximately 0–10% and 25–40%, respectively (Figure 1). The first sample of MS was significantly more toxic to *Chlorella* sp. than the second sample, with IC_25_s of 60 and 91%, respectively (ANCOVA, *P* = 0.008; Table 2). Comparatively, toxicity of both MS samples in the current study appeared lower than that tested by Harford et al. [8] (Figure 1).

**Table 2:** Summary of the toxicity of mine (MS) and synthetic (SS) seepage water from SNM to *Chlorella* sp and *Moinodaphnia macleayi*.

Species/samplenumber/seepage type		Toxicity^a^					
		IC_10_ b		IC_25_		IC_50_	
		% dilution	EC (µS/cm)	% dilution	EC (µS/cm)	% dilution	EC (µS/cm)
*Chlorella* sp.							
Sample 1	MS^c^	37 (31–43)^d^	990(852–1130)	60 (54–66)	1520(1380–1660)	>100	>2320
	SS^c^	47 (39–58)	1180(1000–1430)	>100	>2320	>100	>2390
Sample 2	MS	60 (46–69)	1970(1550–2240)	91 (73-NC^e^)	2900(2360-NC)	>100	>3090
	SS	>100	>3060	>100	>3060	>100	>3060
*Moinodaphnia macleayi*							
Sample 2	MS	13 (8–14)	534(362–568)	15 (10–18)	602(431–705)	21 (17–26)	807 (670–978)
	SS	8 (2–26)	357(156–959)	25 (9–30)	925(390–1090))	34 (26–38)	1230 (959–1360)

Toxicity estimates are presented as percent seepage water as well as electrical conductivity (EC).

a Median inhibition concentrations (IC_50_) not reported as all effects were less than 50%.

b IC_10_, IC_25_, IC_50_: concentration resulting in a 10%, 25% and 50% inhibition of response relative to the control, respectively;

c MS: mine seepage; SS: synthetic seepage.

d Values in parentheses represent 95% confidence limits.

e NC: Not calculable.

Synthetic seepage spiked with Br (220 µg/L), Mn (440 µg/L) and Sr (1400 µg/L) had a similar effect on algal growth as SS without these metals (Table 3). As with the above MS versus SS experiments, there was a similar difference between the effect of SS and MS on algal growth (Table 3). Algal cell counts using microscopy and the electronic particle counter were consistent across most treatments, except treatments exposed to SS, where the microscope counts were higher than the electronic counts (Table 3). Algal cell morphology, in terms of both cell size and aggregation, varied between treatments. Undiluted MS had similar levels of cell aggregation but slightly smaller cells to the control (Figure S1a and S1d). Cells in the two SS treatments also had smaller cells than the control but, unlike the MS treatment, they tended to aggregate (Figure S1b and S1c). The aggregation may explain the slightly lower electronic cell counts compared to microscope counts (Table 3).

**Table 3:** Effect of unmodified synthetic seepage, synthetic seepage with Mn, Br and Sr added, and mine seepage on growth rate of *Chlorella* sp.

Water type	*Chlorella* sp. growth rate (doublings/day; mean ± SEM)	
	By electronic counter	By microscope
Control (Magela Creek water)	1.55±0.02	1.53±0.05
Unmodified synthetic seepage	1.54±0.03	1.73±0.04
Synthetic seepage + 220 µg/L Br, 440 µg/L Mn and 1400 µg/L Sr	1.54±0.01	1.83±0.11
Mine seepage	1.05±0.02	1.03±0.08

Results are presented based on both electronic cell counting (Coulter Counter) and microscope cell counting.

**Figure 2:**
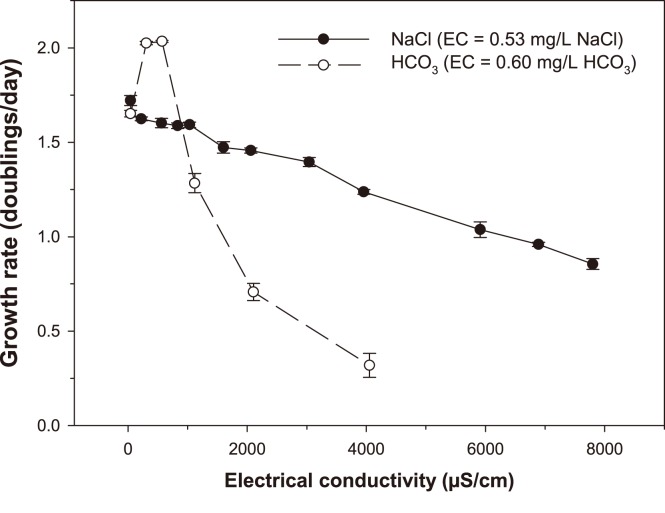
Effect of NaCl and HCO_3_ on growth rate of *Chlorella* sp. Results are expressed as mean ± standard error (n = 3 per tested concentration). Data for NaCl toxicity are pooled from two tests. Mean (% coefficient of variation) (n = 3) control growth rates (expressed as doublings/day) were as follows: NaCl–1.65 (1.82%) and 1.72 (2.69%); HCO_3_–1.65 (1.82%).

The toxicities of NaCl and HCO_3_ to *Chlorella* sp. are shown in Figure 2. The concentration-response relationship for NaCl was linear and gradual, with IC_10_, IC_25_ and IC_50_ values (95% confidence limits) of 620 (40–900) mg/L, 1920 (1630–2130) mg/L and 4,160 (3,790–not calculable) mg/L NaCl, respectively. The NaCl IC_10_, IC_25_ and IC_50_ values corresponded to EC values of 1200 µS/cm, 3600 µS/cm and 7750 µS/cm, respectively. The concentration-response relationship for HCO_3_ exhibited a strong algal growth stimulation at 280 and 550 mg/L HCO_3_, followed by a rapid toxicity response thereafter, with an IC_50_ (95% confidence limits) of 510 (440–590) mg/L, corresponding to an EC of 910 µS/cm.

#### 3.2.2 *Moinodaphnia macleayi*

**Figure 3:**
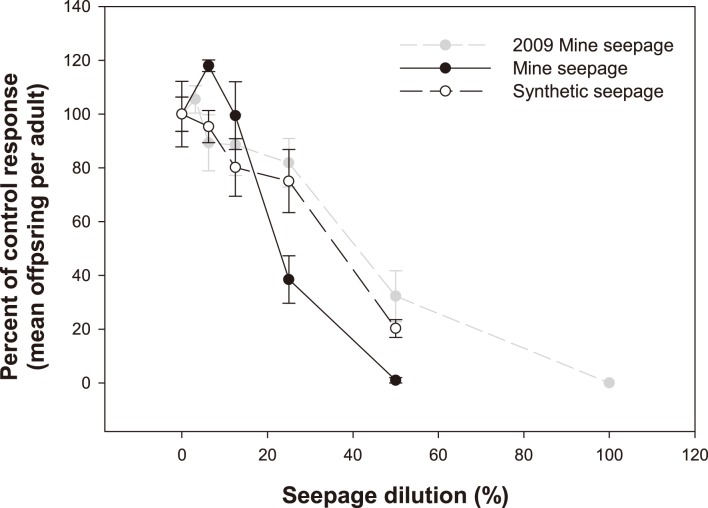
Effect of NaCl and NaSO_4_ on the reproduction of *M. macleayi*. Results are expressed as the mean ± standard error (n = 5 or 10 individuals). Data for NaCl toxicity are pooled from two tests.

Synthetic seepage and MS had a similar effect on *M. macleayi* reproduction (Figure 3, Table 2). Although the extent of effect on reproduction varied somewhat at the 25% and 50% seepage treatments (Figure 3), the overall concentration-response relationships for MS and SS were not significantly different (ANCOVA, *P* = 0.208). Toxicity of the MS in the current study appeared slightly higher than that tested by Harford et al. [8] (Figure 3).

**Figure 4:**
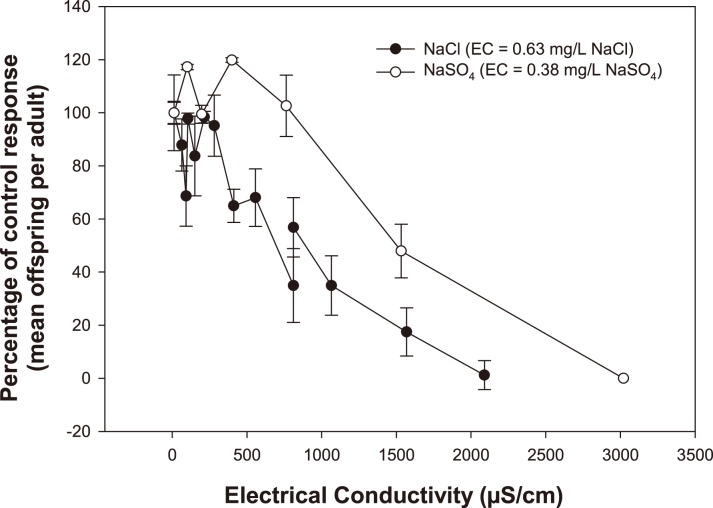
Effect of mine (MS) and synthetic (SS) seepage on 3 brood reproduction of *Moinodaphnia macleayi*. Results are normalized to the percent of control response, and are expressed as mean ± standard error (n = 10 per tested concentration). Mine seepage toxicity data from Harford et al. [8] are also plotted. Mean ± standard error (n = 10) control offspring per adult and percent adult survival for the present study were as follows: MS–20±2.4, 80% survival; SS–34±2.3, 90% survival.

The toxicities of NaCl_2_ and NaSO_4_ to *M. macleayi* are shown in Figure 4. Compared to the NaSO_4,_ the NaCl_2_ had higher toxicity with IC_10_, IC_25_ and IC_50_ values (95% confidence limits) of 20 (6–100) mg/L, 125 (100–250) mg/L, and 250 (170–350) mg/L NaCl, respectively. The NaCl IC_10_, IC_25_ and IC_50_ values corresponded to EC values of 80 µS/cm, 400 µS/cm and 800 µS/cm, respectively. Compared to NaCl, the concentration-response relationship for Na_2_SO_4_ showed a parallel gradient of reproduction decrease but with a higher no-effect threshold. The Na_2_SO_4_ IC50 (95% confidence limits) was 550 (480–640) mg/L, corresponding to an EC of 1420 µS/cm.

**Figure 5:**
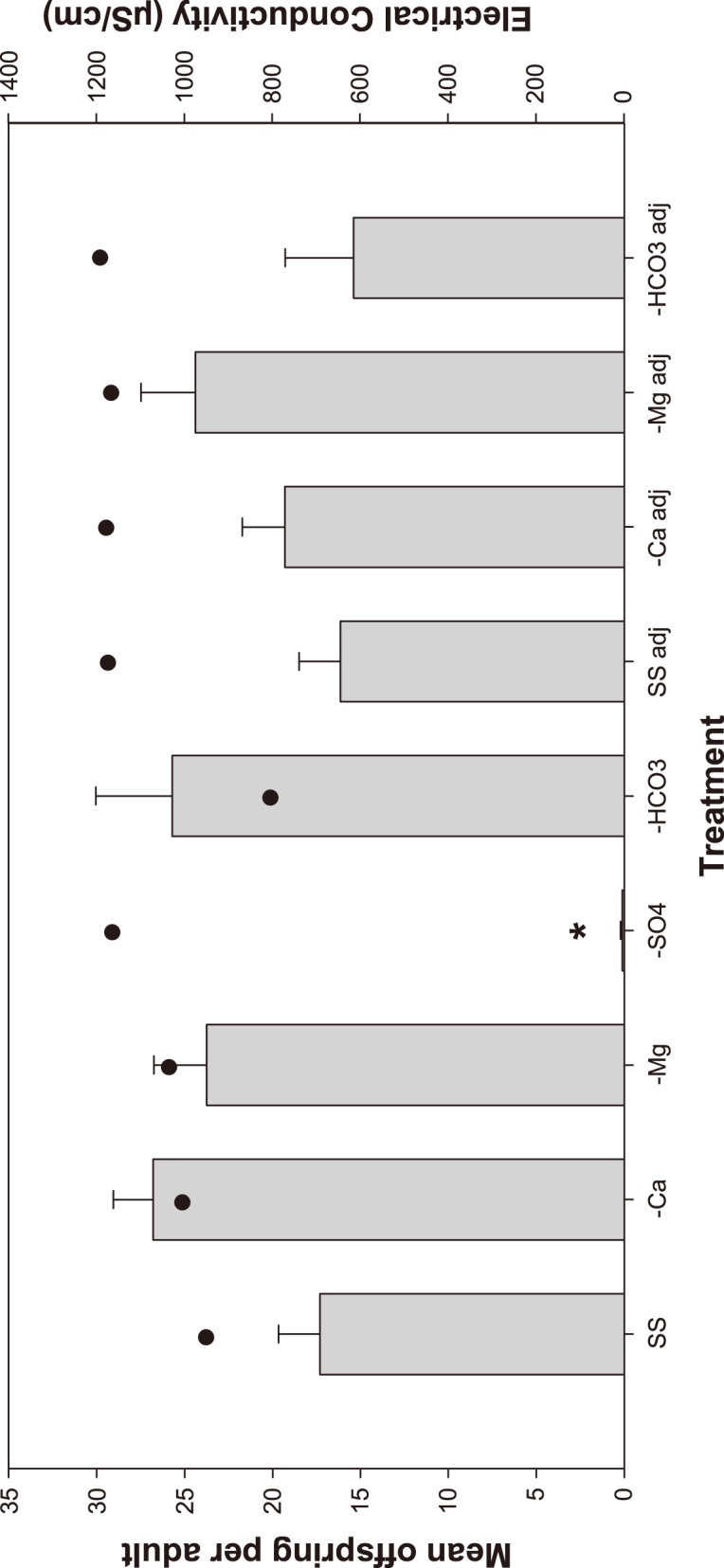
Effect of unmodified synthetic seepage (SS) and synthetic seepage with various ion excluded (–Mg, –Ca, –SO_4_, and –HCO_3_) on 3 brood reproduction of *Moinodaphnia macleayi*. The secondary axis shows the electrical conductivity of the individual waters. Data are pooled from two toxicity test and results are expressed as the mean ± standard error (n = 19–20 individuals, except for –HCO_3_ n = 10). * indicates the –SO_4_ treatment is statistically different from the SS treatment (*P* = 0.008).

Removing specific ions from the SS, and replacing them with other major ions, resulted in some significant changes in toxicity (Figure 5). The most notable effect was complete mortality of *M. macleayi* in the treatment with SO_4_ excluded, in which the cations were replaced with the addition of MgCl_2_ and CaCl_2_. This treatment also had the highest EC of 3200 µS/cm (Figure 5). In comparison with the SS, the synthetic waters with Ca, Mg and HCO_3_ increased the *M. macleayi* reproduction, although these increases were not significantly different to the SS. Increasing the EC of the synthetic waters to a consistent level of 1200 µS/cm reduced the reproduction in the –Ca and –HCO_3_ groups but not the –Mg or SS. This reduced reproduction was most pronounced in the –HCO_3_ treatment. Forward stepwise regression of the results indicated that Cl (P = 0.003) and SO_4_ (P = 0.015) were significant predictors of *M. macleayi* reproduction.

## Discussion

### 4.1 Comparison of seepage toxicity between 2009 and 2011

A comparison was conducted to confirm that the toxicity of the seepage to *Chlorella* sp. and *M. macleayi* in 2011 was comparable to that observed in 2009 by Harford et al. [8]. Water chemistry between the 2009 and 2011 seepage samples was similar, albeit across a relatively wide EC range of approximately 2000 to 3000 µS/cm. The ionic composition in both years (and between both batches in 2011) was dominated by SO_4_, Mg and Ca. The toxicity of MS to *M. macleayi* appeared to be higher in 2011 than the 2009 seepage (Figure 3). This different toxicity may be attributed to the difference in ionic concentration between the seepage waters (i.e. 2009 EC of 2300 µS/cm; 2011 EC of 3100 µS/cm).

In contrast to the results for *M. macleayi*, the 2011 seepage appeared to be less toxic to *Chlorella* sp. relative to the 2009 seepage (Figure 1). Exposure to the whole (100%) seepage waters from 2011 resulted in a 42% (sample 1) and 25% (sample 2) reduction in growth rate of *Chlorella* sp. compared with 56% for the 2009 seepage [8]. Notwithstanding the differences in seepage toxicity between 2009 and 2011, the responses of both species were, in an overall sense, comparable, in that significant effects were observed, ranging from a partial (i.e. <50%) effect for *Chlorella* sp. to a full (i.e. 100%) effect for *M. macleayi*. Thus, the seepage water in the present study was appropriate to assess the hypothesis originally proposed by Harford et al. [8].

### 4.2 Comparison of MS and SS toxicity and possible causes of toxicity

It was hypothesised that the toxicity of the SNM seepage water was due to its major ion constituents. The approach of comparing the toxicity of a mine seepage (MS) to that of a synthetic seepage (SS) simulating only the major ion composition of the MS has been successfully used in other studies to assess the contribution of major ions/salinity to effluent toxicity [4], [6], [13], [14]. The rationale is that if the toxicity of the synthetic water simulating the major ion composition matches that of the whole effluent, then it can be concluded that the majority if not all of the toxicity is being caused by the major ions.

For *Chlorella* sp., the toxicities of SS and MS were significantly different, with SS exhibiting very little if any toxicity, and undiluted MS exhibiting 25–40% reductions in algal cell growth. On this basis, the hypothesis that the toxicity of the seepage water is solely due to its major ion concentrations could not be supported for *Chlorella* sp. Subsequently, the difference in the toxicity of SS and MS to *Chlorella* sp. was investigated by adding Br (220 µg/L), Mn (440 µg/L) and Sr (1400 µg/L) to SS, which were found in MS at concentrations well above the natural background. However, their addition to SS did not increase its toxicity to *Chlorella* sp. and, as such, the toxicity of MS could not be attributed to these metals. The water quality data provided few additional clues on the difference in toxicity between SS and MS.

Apart from the initial 10–20% growth stimulation of *Chlorella* sp. at the lower MS concentrations (probably due to HCO_3_; see Section 4.5), the response of *Chlorella* sp. to MS sample 2 was very similar to the alga’s response to NaCl, when the ion concentrations were expressed as EC (Figure S3). In contrast to the difference between SS and MS toxicity, this similarity suggests a general salinity effect. However, MS sample 1 was more toxic than MS sample 2 and as would be predicted for a general salinity effect based on NaCl toxicity (as EC; Figure S3), despite having lower concentrations of Ca, Mg and SO_4_ ions and also lower EC. This suggested another source of toxicity or a factor increasing the salinity/major ion toxicity in MS sample 1, and also highlighted the difficulty of using EC as a predictor of toxicity for *Chlorella* sp.

During the algal toxicity tests, algal cell morphology appeared different between MS and SS treatments. Specifically, size histograms from the electronic particle counter often showed bimodal distributions in the Coulter Counter histograms, with a larger cell size peak in addition to the typical peak at ∼4.5 µm, suggested a significant change in cell morphology in some of the treatments (Figure S2). Microscope observations indicated that this was due to the aggregation of algal cells into large clumps of >5 cells (Figure S1). Aggregation is known to occur in high ionic strength media, due to changes in the surface charge of the algal particles, causing them to be attracted to each other [15]. However, the aggregation in this study occurred consistently in the 50 and 100% SS treatments but did not occur in the 100% MS treatments, despite the MS and SS treatments having the same ionic strength. Algal aggregation can also occur when algae are stressed and produce sticky exudates [16], which might explain the inconsistent aggregation in MS. It is difficult to identify the exact cause of the aggregation, but the factor(s) involved may also be related to the differences in toxicity between MS and SS.

In contrast to *Chlorella* sp., the toxicities of SS and MS to *M. macleayi* were found to be statistically similar. Consequently, the hypothesis that the toxicity of the seepage water is solely due to its major ion concentrations was supported for *M. macleayi*. Additionally, the concentration-response relationship for the MS and SS also closely resembled that of the NaCl and the NaSO_4_ toxicity tests (Figure S4), which suggested that the composition of the salt was of less importance for this species and that a general salinity was the cause of the effects. This prompted an experiment to assess whether any specific ions or ion combinations could be identified as the key contributors to toxicity. Harford et al. [8] discounted ions such as K, Cl, Na and Ca contributing to the toxicity of MS, and suggested that Mg and SO_4_ were the potential candidates. Additionally, as high Ca concentrations are known to reduce the toxicity of Mg [17] and SO_4_ [18]–[20], then its presence in MS may be preventing or reducing MS toxicity. Consequently, the ion exclusion experiment for *M. macleayi* focused on the exclusion of Mg, SO_4_ and Ca from the SS. The results showed that removal of SO_4,_ and replacement with Cl, caused a significant increase in the toxicity of SS and complete mortality of all *M. macleayi*. Removal of Ca and Mg, and replacement with Na, increased the reproduction of *M. macleayi* suggesting that Na was less toxic than both Mg and Ca. Magnesium being higher in toxicity than Na concurs with Mount et al. [5] but they also concluded that Ca was less toxic, which is different to what the *M. macleayi* result suggests. Removal of HCO_3_ from the SS and replacement with SO_4_ resulted in increased rates of reproduction. However, it should also be noted that throughout the toxicity test, the pH of the –HCO_3_ treatment tended to drift lower than the starting pH of 8.0. This was drift was highest on the first day and decreased by 0.4 pH units. The lower pH may have improved the performance of the *M. macleayi* as they usually inhabit waters with a pH<7. Hence, the effect of this treatment may have been confounded and it was not included in the stepwise regression.

Toxicity estimates converted to EC and compared to the effects observed from the NaCl single salt toxicity tests accurately predicted the effect of the SS with only a 7% difference (Table 4). Conversely, the effects observed in the NaSO_4_ single salt toxicity tests did not predict the effect of the SS with a 32% difference. This concurs with the stepwise regression, which identified Cl as a better indicator of toxicity than SO_4_. Excluding the SO_4_ also resulted in complete mortality of the organisms, which indicates that equivalent concentrations of Cl were more toxic than SO_4_. Comparing the results of the modified synthetic waters with the results of the NaCl and NaSO_4_ toxicity tests showed no obvious pattern, which shows that EC is not the sole factor affecting toxicity (Table 4).

**Table 4:** Comparison of single salt toxicity estimates with observed effect of synthetic waters.

Synthetic water a	ElectricalConductivity(µS/cm)	% effectobserved	% EffectPredictedfrom NaCl	Difference	Effect predictedfrom NaSO_4_	Difference
SS	950	56	62.5	–7	24	32
–Ca	1004	31	65	–34	29	2
–Mg	1034	39	66	–27	30	9
–SO_4_	1163	100	71	29	40	60
SS adj	1173	58	71	–13	34	24
–Ca adj	1177	50	71	–21	34	16
–Mg adj	1166	37	71	–34	33	4

a SS = Synthetic Seepage, –Ca = SS with no calcium, –Mg = SS with no magnesium; –SO_4_, adj = treatments adjusted with sodium chloride to balance EC.

Forward stepwise regression of the results indicated that Cl (P = 0.003) was the primary cause of toxicity but this was driven primarily by the high reproduction in the controls which had low concentrations of Cl and the 100% reduction in the –SO_4_ synthetic water. The –SO_4_ treatment had high Cl because this anion was used to replace the SO_4_ in that synthetic water. The stepwise regression result may also have been influenced by the use of NaCl to increase the osmolarity of the adjusted treatments. Increasing the EC of the synthetic solutions reduced the reproduction of all treatments, except the adjusted SS treatment, which produced the same response as the unadjusted treatment despite needing a higher amount of NaCl addition compared to other treatments. In the end, Cl was not present in the seepage water at high concentrations and was only present at significant concentrations in treatments due to the manipulation of the synthetic waters. However, the forward stepwise regression also indicated SO_4_ as a predictor for *M. macleayi* reproduction, which is probably due to it being the most dominant ion present in the synthetic waters and also appeared to be driven by high reproduction in waters with low SO_4_ concentrations, i.e. MCW control. Including EC as a variable in the forward stepwise regression concluded that EC is a more significant predictor of *M. macleayi* reproduction than SO_4_, which appears to be driven by high reproduction in the low EC control waters. These results indicate a general salinity effect with major ions contributing to toxicity to differing degrees when the ionic composition of the waters changed.

### 4.3 Other major ion studies for *Chlorella* sp. and *M. macleayi*

Few studies have reported the toxicity of NaCl to *Chlorella* sp. Chimiklis and Karlander [21] assessed the effects of NaCl at varying light conditions and in the absence and presence of Ca on *Chlorella sorokiniana*. At background Ca concentrations (1 mg/L) and base light conditions (∼1.5 mw/cm^2^), algal growth rate was reduced by 15%, 30% and 65% relative to controls at approximately 6 g/L, 12 g/l and 18 g/L, respectively. Increasing Ca concentration and light intensity reduced NaCl toxicity further. The toxicity of NaCl to *Chlorella* sp. in the present study was somewhat higher than that reported by [21] with IC_10_ and IC_50_ values of approximately 0.7 g/L (1200 µS/cm) and 4.2 g/L NaCl (7700 µS/cm), respectively. The most likely explanation for the difference in NaCl toxicity between the two species is the different test media used. The toxicity of NaCl to *C. sorokiniana* was assessed in a highly nutrient-enriched medium, compared to a dilute medium (with only minimal NO_3_ and PO_4_ added) for *Chlorella* sp. in the present study.

As with *Chlorella* sp., there are few existing data on the toxicity of salts to *M. macleayi*. Mohammed and Agard [22] compared the toxicity of NaCl to *M. macleayi* and several other cladoceran species. They reported a 48 h LC_50_ for *M. macleayi* of 1.5‰ (i.e. 1.5 g/L) NaCl, which represented the second most sensitive result. In comparison, the present study reported a 50% reduction in *M. macleayi* (3 broods) reproduction at approximately 0.25 g/L (800 µS/cm). Given the toxicity value from Mohammed and Agard [22] represents an acute value (compared to a chronic value for the present study), it can be reasonably concluded that the sensitivity of *M. macleayi* between the two studies was similar.

### 4.4 Sulfate toxicity

Harford et al. [8] suggested that SO_4_ may have been contributing to seepage toxicity. However, when SO_4_ was excluded, toxicity increased, possibly due to the addition of Cl along with the Mg and Ca counter ions. The toxicity of SO_4_ to *M. macleayi* has not been previously assessed but this study found that SO_4_ was less toxic than Cl. Recently, Elphick et al. [20] assessed the toxicity of SO_4_ in waters of varying water hardness. At a high water hardness of 160 mg/L (as CaCO_3_), the chronic IC_50_ for *C. dubia* (3 brood reproduction) was 1257 mg/L. However, at higher water hardness (360 mg/L), the IC_50_ decreased to 843 mg/L. This was thought to be due to the toxic effects of EC/salinity, rather than SO_4_ alone, becoming increasingly dominant [20]. Thus, given the water hardness of the MS and SS (∼900 mg/L as CaCO3) was much higher than that assessed by Elphick et al. [20], this lends further support to the notion that the overall EC/salinity of the MS is the key cause of toxicity.

### 4.5 Bicarbonate toxicity to *Chlorella* sp

Bicarbonate stimulated growth of *Chlorella* sp. at low levels (up to ∼250 mg/L) and started to have a toxic effect beyond this concentration, with an IC_50_ of 510 mg/L (910 µS/cm). The initial growth stimulation was likely to be due to the fact that HCO_3_ can be used as a source of CO_2_ to photosynthetic organisms such as *Chlorella* sp. [23]. The small growth stimulation measured for *Chlorella* sp. in all MS and SS samples (see Figure 1) may have been due to the presence of HCO_3_. In this study, it was noted that EC may not be a reliable reflection of an effluent’s toxicity. This is particularly true in the case of single salt toxicity estimates. At similar ECs, NaCl and NaHCO_3_ had significantly different effects on *Chlorella* sp., highlighting the notion that while EC might be a key cause of toxicity, ECs of various saline water types should not be relied solely upon as predictors of toxicity.

## Conclusions

For *Chlorella* sp., the toxicity of the seepage water was not solely due to its major ion concentrations because there were differences in effects caused by the mine seepage and synthetic seepage. However, for *M. macleayi* the hypothesis that toxicity was due to major ion concentrations was supported because similar effects were observed following exposure to MS and SS. Sulfate was identified as a major ion that could predict the toxicity of the synthetic waters, which might be expected as it was the dominant major ion in the seepage water. Despite a relationship between sulfate and toxicity, it is unlikely that sulfate was the primary cause of toxicity in the seepage water as EC was a better predictor of the observed effects. Ultimately, the results show that no specific major ion clearly drives the toxicity of saline seepage waters and the effects are probably due to the overall EC of the waters.

## Supporting Information

Figure S1 **Images showing various morphological changes, notably aggregations in B and C, occurring to the** ***Chlorella*** **sp. cells in different water types (>400×magnification).** A–Magela Creek Water (MCW); B–Synthetic Seepage (SS); C; Synthetic Seepage with Br, Mn and Sr added (SS (+ Br, Mn, Sr)); and D–Mine Seepage (MS).
(PDF)

Figure S2 **Examples of electronic particle counter histograms for** ***Chlorella*** **sp. exposed to a) Control b) Synthetic Seepage c) Mine Seepage for 72 hours.**
(PDF)

Figure S3 **Response of** ***Chlorella*** **sp. growth rate to Mine seepage (samples 1 and 2) and sodium chloride (NaCl), with concentrations expressed as Electrical Conductivity.**
(PDF)

Figure S4 **Comparison of** ***M. macleayi*** **concentration-response relationships for Mine Seepage (MS), Synthetic Seepage (SS), sodium sulfate (NaSO_4_) and sodium chloride (NaCl), with concentrations expressed as Electrical Conductivity.**
(PDF)

Dataset S1 **Summarised raw data.**
(XLSX)
