# Supplementary material for: Identifying the Cause of Toxicity of a Saline Mine Water
Source: PLoS One. 2014 Sep 2;9(9):e106857. doi: 10.1371/journal.pone.0106857 (PMC4152331; doi:10.1371/journal.pone.0106857)
Supplement: Figure S1 — Images showing various morphological changes, notably aggregations in B and C, occurring to the Chlorella sp. cells in different water types (>400×magnification). A–Magela Creek Water (MCW); B–Synthetic Seepage (SS); C; Synthetic Seepage with Br, Mn and Sr added (SS (+ Br, Mn, Sr)); and D–Mine Seepage (MS). (PDF) [file pone.0106857.s001.pdf]

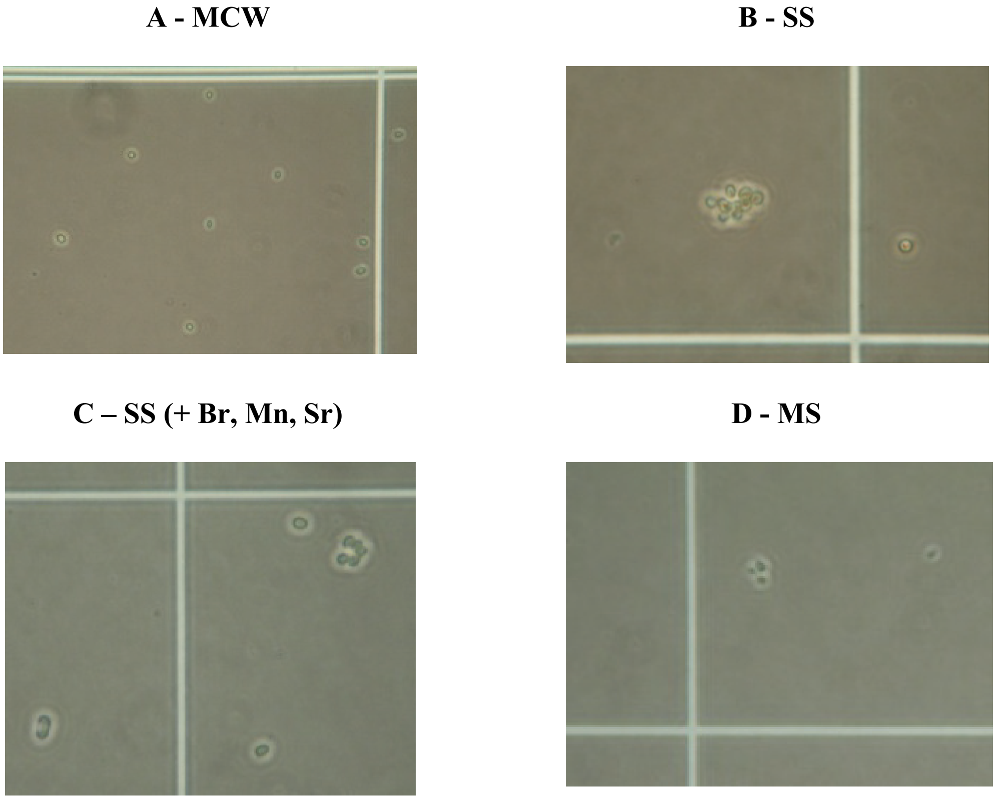

Figure S1 Images showing various morphological changes, notably aggregations in B and C, occurring to the *Chlorella* sp. cells in different water types (>400× magnification). A – Magela Creek Water (MCW); B – Synthetic Seepage (SS); C; Synthetic Seepage with Br, Mn and Sr added (SS (+ Br, Mn, Sr)); and D – Mine Seepage (MS).
