# Supplementary material for: Identifying the Cause of Toxicity of a Saline Mine Water
Source: PLoS One. 2014 Sep 2;9(9):e106857. doi: 10.1371/journal.pone.0106857 (PMC4152331; doi:10.1371/journal.pone.0106857)
Supplement: Figure S2 — Examples of electronic particle counter histograms for Chlorella sp. exposed to a) Control b) Synthetic Seepage c) Mine Seepage for 72 hours. (PDF) [file pone.0106857.s002.pdf]

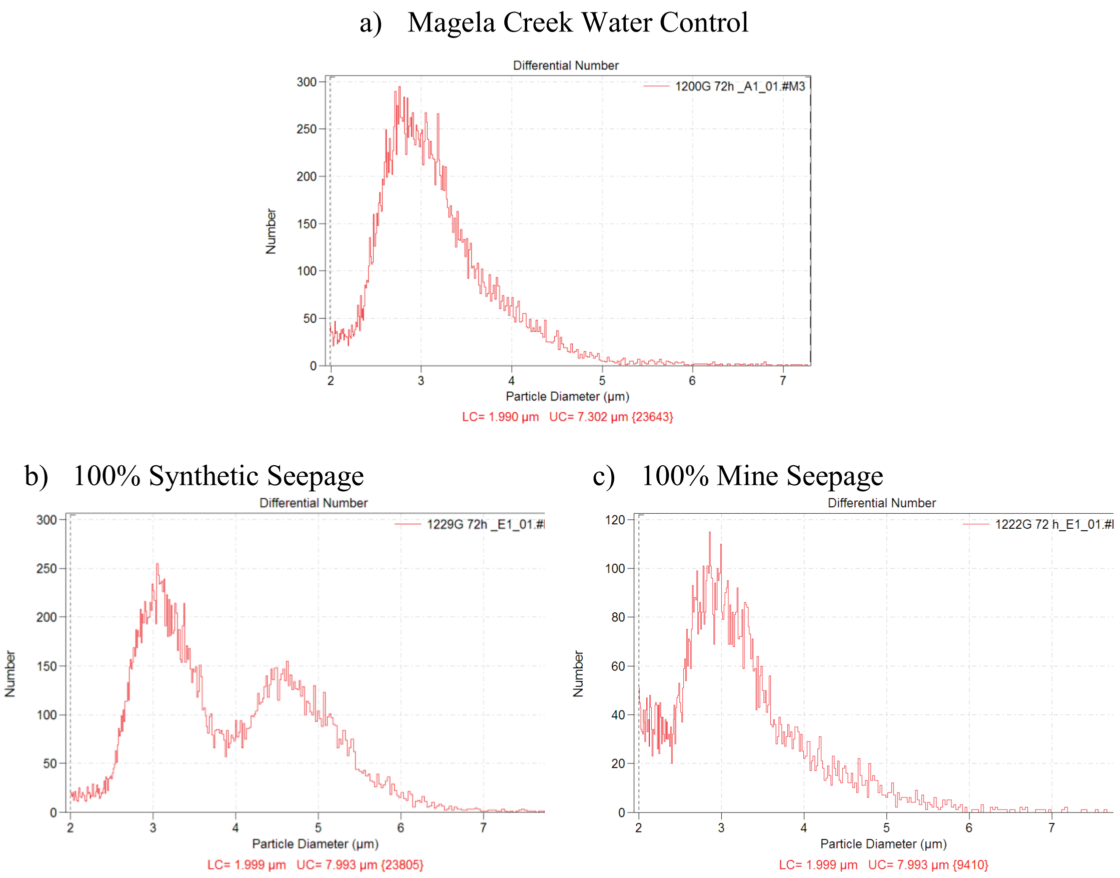

Figure S2 Examples of electronic particle counter histograms for *Chlorella* sp. exposed to a) Control b) Synthetic Seepage c) Mine Seepage for 72 hours.
