# Supplementary material for: Identifying the Cause of Toxicity of a Saline Mine Water
Source: PLoS One. 2014 Sep 2;9(9):e106857. doi: 10.1371/journal.pone.0106857 (PMC4152331; doi:10.1371/journal.pone.0106857)
Supplement: Figure S3 — Response of Chlorella sp. growth rate to Mine seepage (samples 1 and 2) and sodium chloride (NaCl), with concentrations expressed as Electrical Conductivity. (PDF) [file pone.0106857.s003.pdf]

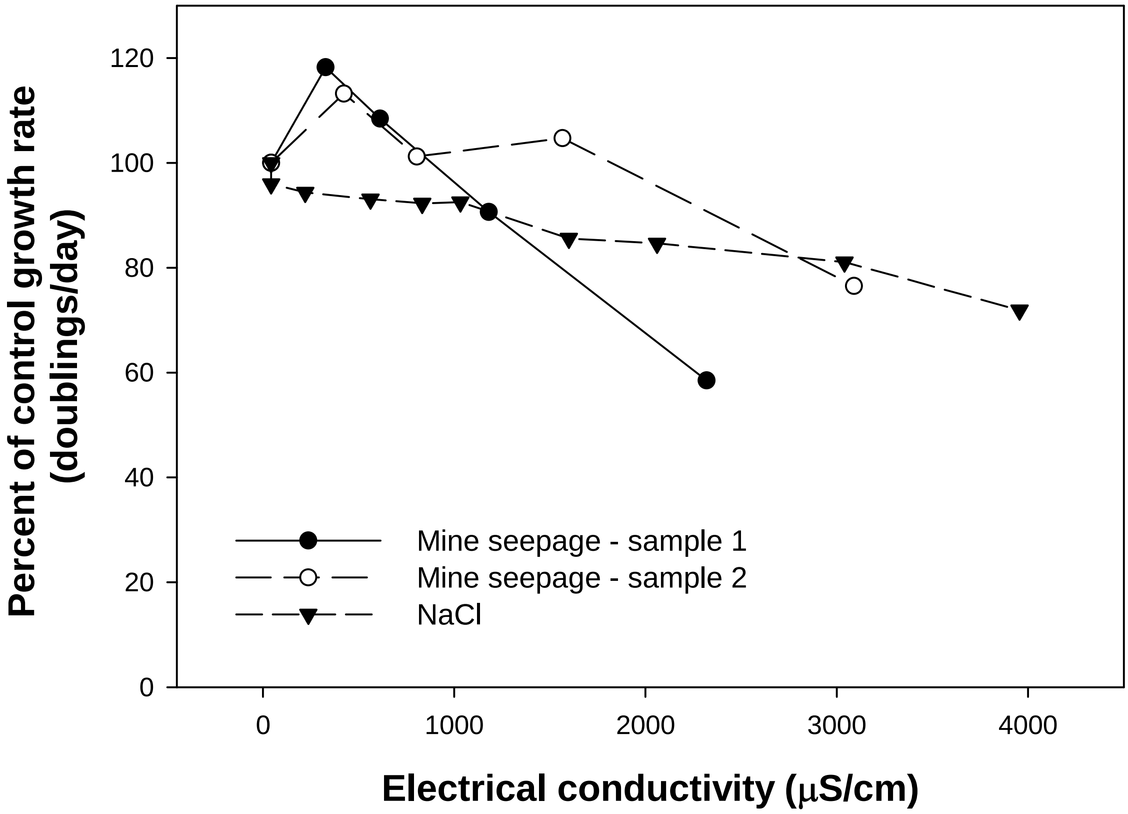

Figure S3 Response of *Chlorella* sp. growth rate to Mine seepage (samples 1 and 2) and NaCl, with concentrations expressed as Electrical Conductivity.
