# Supplementary material for: Identifying the Cause of Toxicity of a Saline Mine Water
Source: PLoS One. 2014 Sep 2;9(9):e106857. doi: 10.1371/journal.pone.0106857 (PMC4152331; doi:10.1371/journal.pone.0106857)
Supplement: Figure S4 — Comparison of M. macleayi concentration-response relationships for Mine Seepage (MS), Synthetic Seepage (SS), sodium sulfate (NaSO4) and sodium chloride (NaCl), with concentrations expressed as Electrical Conductivity. (PDF) [file pone.0106857.s004.pdf]

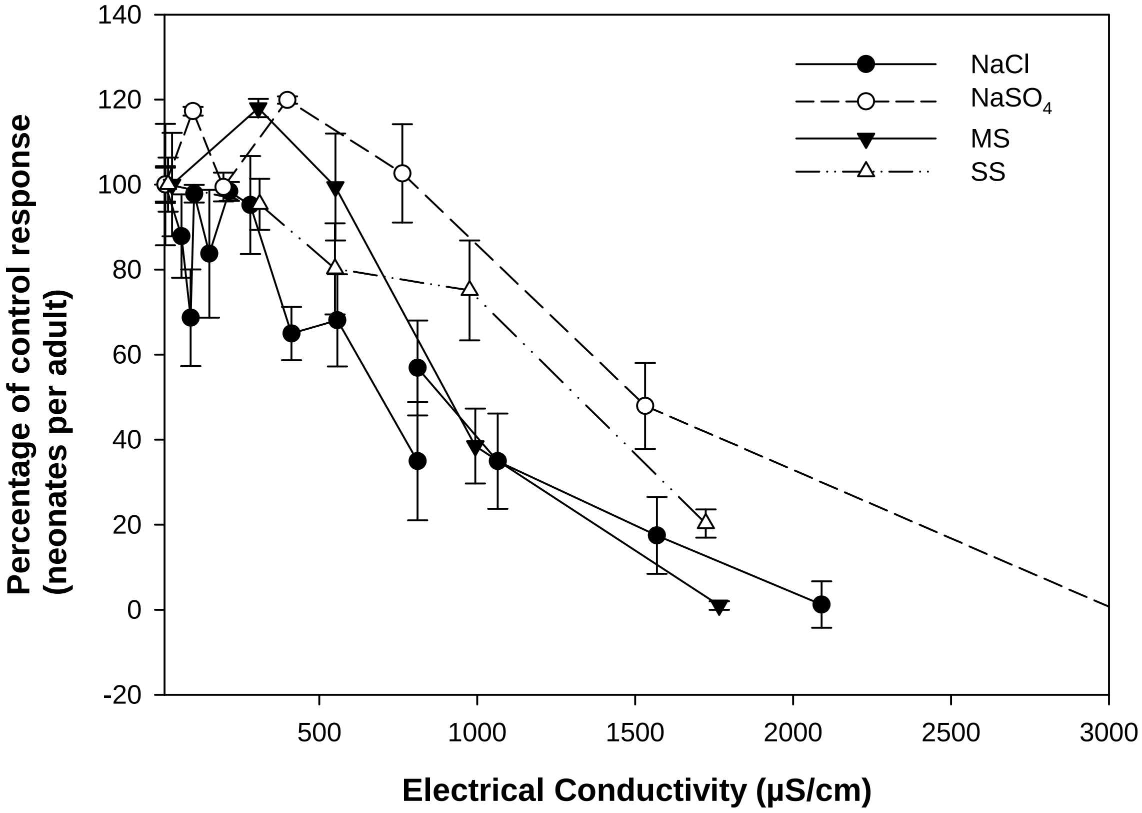

Figure S4 Comparison of *M. macleayi* concentration-response relationships for Mine Seepage (MS), Synthetic Seepage (SS), sodium sulfate ($NaSO_4$) and sodium chloride (NaCl), with concentrations expressed as Electrical Conductivity.
